# Administration of Riluzole Oral Suspension During the Different Stages of Amyotrophic Lateral Sclerosis

**DOI:** 10.3389/fneur.2021.633854

**Published:** 2021-07-08

**Authors:** Mónica Povedano Panades, Philippe Couratier, Katie Sidle, Gianni Sorarù, Georgios Tsivgoulis, Albert C. Ludolph

**Affiliations:** ^1^Unitat Funcional de Motoneurona, Neurofisiologia-Servei de Neurologia Hospital Universitario de Bellvitge-Institut d'Investigació Biomèdica de Bellvitge, Barcelona, Spain; ^2^Centre de Référence Maladies Rares SLA et Autres Maladies du Neurone Moteur, CHU, Limoges, France; ^3^Motor Neuron Disease Association Motor Neuron Disease Care and Research Centre, National Hospital for Neurology and Neurosurgery, London, United Kingdom; ^4^Department of Neurosciences, University of Padova, Padua, Italy; ^5^Second Department of Neurology, “Attikon” University Hospital, National & Kapodistrian University of Athens, Athens, Greece; ^6^Department of Neurology, University of Ulm, Ulm, Germany

**Keywords:** dysphagia, riluzole, oral suspension, Amyotrophic Lateral Sclerosis, swallowing difficulties

## Introduction

Amyotrophic Lateral Sclerosis (ALS) is a fatal adult-onset neurodegenerative disorder that affects the upper and/or lower motor neurons and is associated with the degeneration of cortical and spinal motor neurons (1, 2).

Dysphagia, a swallowing disorder related to tongue atrophy and dysfunction in the closure of the soft palate and the larynx that progressively inhibit the passage of food or liquid from the mouth through the pharynx into the esophagus, is one of the most severe and debilitating symptoms of ALS (3–5). Even though it has an earlier onset and is more severe in ALS bulbar (3) than in spinal patients, it has been demonstrated that more than 30% of spinal onset patients show dysphagia symptoms at diagnosis, and in the advanced stages of the disease of both phenotypes, more evidently, more than 80% of patients manifest bulbar involvement and dysphagia (3, 5). To preserve oral feeding and postpone the need for percutaneous endoscopic gastrostomy (PEG), the first interventions in patients with dysphagia include postural compensation maneuvers, swallowing techniques, and diet adjustments to adapt the consistency of foods and liquids to the patient's impairment (5).

As reported in a retrospective study (5), the prevalence of dysphagia during a mean follow-up of almost 2 years increased from 35.2% at diagnosis to 73.3% at final follow-up in spinal-onset patients, and from 94.7 to 98.2% in bulbar-onset patients (5). Interestingly, six patients with spinal onset (8%) and dysphagia found by FEES (fiberoptic endoscopic evaluation of swallowing) did not report the disorder as symptomatic (i.e., demonstrated silent aspiration). Furthermore, while at the beginning of the observation period 45% of patients were on a normal diet and no patients needed PEG, at the end of the follow-up 63% of patients had modified food consistency, and 20.7% had undergone a PEG. In particular, patients with bulbar onset ALS were given food with modified consistency and underwent PEG more frequently than patients with spinal onset (5).

According to the European guidelines on the clinical management of ALS, timing of PEG should take into account patient characteristics such as bulbar symptoms, malnutrition (weigh loss ≥ 10%), respiratory function (before forced vital capacity <50% of predicted), and patients' general condition (6). As shown in the study of Pena et al. (7), PEG is inserted more precociously in bulbar-onset than in spinal-onset patients (19.3 vs. 29.7 months from symptom onset) while the median survival after PEG replacement is similar in the two groups of patients (7.9 vs. 7.1 months). Furthermore, bulbar-onset patients presented shorter survival (42 vs. 29 months after disease onset) and diagnostic delay (10.3 vs. 12.2 months).

## Riluzole

Riluzole is a synthetic benzothiazole drug indicated to extend life or the time to mechanical ventilation for patients with ALS (8). As stated by international guidelines and clinical recommendations, riluzole is the only drug that has been shown to slow the course of all ALS patients and to increase patient's survival; therefore, treatment should be initiated as early as possible after diagnosis (6, 9). Furthermore, riluzole is still considered the gold standard treatment in clinical trials with new potential agents in which new molecules mandatorily have to be compared with riluzole or as an add-on treatment to riluzole (10).

Data from RCTs (randomized clinical trials) (11–14) and following meta-analysis (15) showed that riluzole, at the recommended daily dosage of 100 mg (50 mg every 12 h), has a significant effect on survival with a gain of 3 months in 50% of patients or a 9% increase in the 1-year survival probability (15). The average life expectancy of patients in the pivotal trial was about 12 months (12). Real-world evidence (16–25), based on retrospective and prospective analysis of large patient databases, also suggest significant improvement of survival and significantly prolonged time to need PEG and ventilator support (23) in riluzole-treated ALS patients compared with those who did not receive riluzole (Table 1), with the most prominent effect in patients who started riluzole administration early in the disease (26). Furthermore, it would seem that patients who were treated longer survived longer (27). Finally, riluzole treatment extends the duration of Stages 1 and 2 of the King's College clinical staging if used early, and the effect was maintained in patients with long-term use and prolongs Stage 4 (28).

**Table 1:** Real-world studies of riluzole with a specific focus on survival data.

**Reference**	**Country**	**Type of study**	**Patients riluzole/no riluzole (*n*)**	**Median survival time riluzole/no riluzole (months)**	**Survival from**
Meininger et al. (16)	FR	Retrospective	356/161	18.4/12.4	Diagnosis
Brooks et al. (17)	US	Retrospective	<1,996: 51/241 ≥1,996: 112/65	<1,996: 58.4/47.7 ≥1,996: >67/49.1	Onset of symptoms
Turner et al. (18)	UK	Prospective	299/349	51/32	Onset of symptoms
Traynor et al. (19)	IRL	Retrospective	149/97	14.3/10.1	Diagnosis
Mitchell et al. (20)	UK	Retrospective	148/327	36.84/27	Diagnosis
Zoccolella et al. (21)	IT	Prospective	73/53	18.3/12.4	Diagnosis
Lee et al. (22)	CHN	Retrospective	698/451	n/a	n/a
Georgoulopoulou et al. (23)	IT	Prospective	133/60	43/31	Onset of symptoms
Knibb et al. (24)	UK	Prospective	260/315	17.7^*^	Diagnosis
Chen et al. (25)	CHN	Prospective	415/1.125	88/61 (beyond quartile 3 vs. control)	Diagnosis

*Modified from (26)*.

* *Based on the total number of cases (575)*.

The main drawback of the tablet formulation of riluzole is that patients with dysphagia or PEG may discontinue treatment due to inability to swallow. Swallowing difficulties and dysphagia can lead to a worsening of therapeutic outcomes due to poor adherence to prescribed medications. Indeed, to overcome the difficulties in swallowing solid drug formulations, more than 25% of dysphagic patients with ALS crush the tablet of riluzole or mix it with food (5). Both practices fall outside the riluzole label indication, and since there is no evidence on the efficacy and safety of crushed tablets, it may have an impact on absorption characteristics, cause dosage error, produce an anesthetic effect on the tongue, and increase the risk of respiratory infections (due to the micro aspiration of particles when a crushed tablet is given mixed, i.e., with yogurt) (4, 5, 29).

## Discussion

The worsening of swallowing deficits in patients with ALS is associated with weight loss, malnutrition, difficulty taking oral medication and an increased risk of choking and aspiration pneumonia (3, 4). Diet adjustments to adapt the consistency of foods and liquids to the patients swallowing impairment play an important role in preserving oral feeding. Indeed, as reported by the International Dysphagia Diet Standardization Initiative (IDDSI) framework, the food texture and thickness of liquids should be modified according to the severity of patients' dysphagia (i.e., the thickness of liquids should be increased, and the food texture should be modified to reduce the risk of penetration/aspiration and/or improve swallowing function).

In general, in terms of liability, it is important not to alter a solid-dose oral formulation, as also reported in the “Five Rights of Medication Administration,” which help reduce the risk of medication errors and require that “the right medicine is given to the right person, at the right time, using the right dose, in the right form” (30). Furthermore, all products should be prescribed and administered in accordance with the manufacturing authorization, and when drugs are used outside their license, the prescriber, dispenser, and/or person responsible for the provision or administration of the medication must accept liability for any adverse effects (31). Indeed, the liability rests with the pharmaceutical manufacturer only if a medicine is prescribed in the patient group, at the recommended dose, and *via* the recommended route of administration, in the form in which it was tested (32).

According to the algorithm reported in the Consensus guideline on the medication management of adults with swallowing difficulties (31), when the swallowing difficulty is likely to be long-term— as in the case of patients with ALS—and the oral route is appropriate, it is recommended to check whether a licensed liquid is available or dispersible formulation with a suitable consistency in order not to discontinue the treatment.

Finally, since changing the formulation of a product may alter its bioavailability, efficacy, and/or side-effect profile, it is recommended to check dose equivalence and evaluate efficacy and side effects frequently (31). Altering a solid-dose formulation should be considered only as a last option and practiced only after a pharmacist has confirmed its safety. Similarly, according to the practical guide of the British Association for Parental and Enteral Nutrition, crushing tablets and opening capsules for the administration *via* feeding tubes should be considered as a last resort since this practice falls outside the drug's license and can also cause tube blockage (33). When the administration route *via* feeding tube is the only available route, a liquid formulation should be used. The consistency of the liquid formulation (in order to avoid tube blockage) and its composition in terms of excipient contents should be carefully considered (34).

In order to ensure therapeutic continuity, an oral suspension of riluzole has been developed. It has been formulated as a 5 mg/ml suspension, which provides the usual dose of riluzole in a total volume of 10 ml, and may allow patients with dysphagia (or at risk of developing it) and patients with PEG to continue riluzole treatment for longer (4).

The availability of an oral suspension of riluzole offers clear and important advantages over tablets: first of all, it is a homogenous dispersion of riluzole designed with a novel flocculation technology to minimize the local anesthetic effect of the drug in the mouth and the unpleasant metallic taste. Its texture is classified as a 2-nectar-like texture (as classified by IDDSI), which is the most appropriate texture for swallowing difficulties, so it helps to avoid micro-aspirations, offering easy and safety swallow and administration to patients. Additionally, riluzole 5 mg/ml oral suspension is bioequivalent to riluzole 50 mg tablets (35) and is ready to use, without external manipulation or premixing, providing a more accurate dosing and enhanced compliance (4, 29, 36).

Riluzole oral suspension is also suitable for administration *via* enteral feeding tubes since it is a liquid formulation with the appropriate consistency to avoid PEG obstructions and with the appropriate contents of sorbitol to avoid gastrointestinal problems (37, 38). Moreover, as reported by Brooks et al. (39) riluzole oral suspension is bioequivalent when administered intragastrically and orally and both administration methods are well-tolerated. Thanks to these results rilzuole oral suspension is authorized both for oral administration and *via* PEG-tubes (40). This is an important difference from tablets, because riluzole oral suspension is the only formulation that can be safely administered even to patients who require PEG insertion.

In this opinion article we suggest the use of riluzole oral suspension, since it overcomes the limitations associated with tablet formulation with a particular benefit for patients with dysphagia. Moreover, riluzole oral suspension is particularly indicated for patients who have undergone PEG placement. Starting therapy with riluzole oral suspension rather than tablets could ensure better compliance, minimizing adherence losses and the psychological burden for patients and caregivers due to medication switches.

## Author Contributions

All authors listed have made a substantial, direct and intellectual contribution to the work, and approved it for publication.

## Conflict of Interest

MP reports non-financial support from Italfarmaco and Biogen, outside the submitted work. PC reports grant from Cytokinetics, outside the submitted work. KS reports consultant fee from Italfarmaco, outside the submitted work. GT reports personal fees from Sanofi, Biogen, Italfarmaco, Bayer, Genesis Pharma, CSL Behring, Allergan, and Medtronic, outside the submitted work. The remaining authors declare that the research was conducted in the absence of any commercial or financial relationships that could be construed as a potential conflict of interest.
